# Magnetically actuated swimming and rolling erythrocyte-based biohybrid micromotors†

**DOI:** 10.1039/d3ra05844a

**Published:** 2023-10-23

**Authors:** Qi Wang, Sophie Jermyn, David Quashie, Sarah Elizabeth Gatti, Jaideep Katuri, Jamel Ali

**Affiliations:** a Department of Chemical and Biomedical Engineering, FAMU-FSU Collee of Engineering Tallahassee Florida 32310 USA jali@eng.famu.fsu.edu; b National High Magnetic Field Laboratory Tallahassee Florida 32310 USA; c Department of Biomedical Engineering, Vanderbilt University College of Engineering Nashville Tennessee 37235 USA

## Abstract

Erythrocytes are natural multifunctional biomaterials that can be engineered for use as micro robotic vectors for therapeutic applications. Erythrocyte based micromotors offer several advantages over existing bio-hybrid micromotors, but current control mechanisms are often complex, utilizing multiple external signals, such as tandem magnetic and acoustic fields to achieve both actuation and directional control. Further, existing actuation methods rely on proximity to a substrate to achieve effective propulsion through symmetry breaking. Alternatively, control mechanisms only requiring the use of a single control input may aid in the translational use of these devices. Here, we report a simple scalable technique for fabricating erythrocyte-based magnetic biohybrid micromotors and demonstrate the ability to control two modes of motion, surface rolling and bulk swimming, using a single uniform rotating magnetic field. While rolling exploits symmetry breaking from the proximity of a surface, bulk swimming relies on naturally occurring shape asymmetry of erythrocytes. We characterize swimming and rolling kinematics, including step-out frequencies, propulsion velocity, and steerability in aqueous solutions using open-loop control. The observed dynamics may enable the development of future erythrocyte micromotor designs and control strategies for therapeutic applications.

## Abstract 2

Using a single rotating magnetic field, RBC biohybrid micromotors can be controlled to achieve propulsion *via* swimming and rolling modes. The propulsion mechanism, directional control, and behavior in different fluids is investigated.

## Graphical abstract

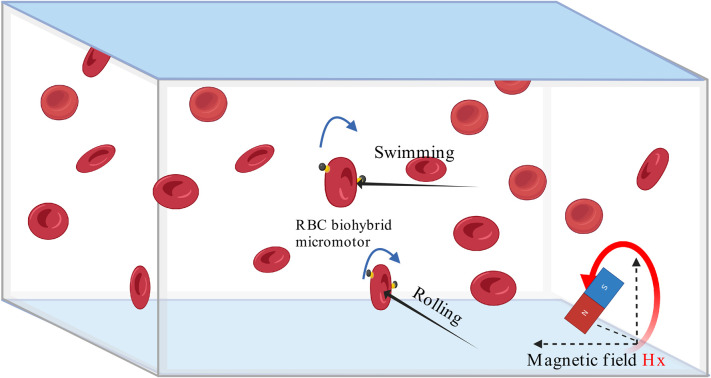

## Introduction

Micromotors are small devices that can transduce local energy into directed propulsion. A common strategy to develop these systems has been to rely on biologically inspired designs such as helical micromotors which derive their shape from bacterial flagella but are made of synthetic materials and are actuated by rotating magnetic fields.^1^ Similarly, chains of magnetic colloidal particles have been assembled *via* DNA-linking and actuated by oscillating magnetic fields to achieve propulsion inspired by beating flagella.^2^ Alternatively, synthetic micromotors have been developed that are propelled by chemical, electrical, or acoustic gradients and a number of applications ranging from drug delivery, environmental remediation and sensing have been demonstrated.^3^ A recent effort in this area is towards developing bio-hybrid micromotors that combine biological and non-biological components. These micromotors have unique advantages such as low toxicity and offer novel properties arising from the combination of synthetic and biological material that are inaccessible in purely biological or synthetic systems.^3–7^ Erythrocytes (red blood cells, RBCs) are promising materials to form the biological component of bio-hybrid micromotors due to their inherent biocompatibility and unique properties of their membranes that can be easily modified to externally couple with synthetic components and internally loaded through controlled opening and closing of nanopores.^8–10^ As early as 1973, RBCs have been used as therapeutic cargo carriers, loaded with enzymes (β-glucosidase and β-galactosidase) by rapid hemolysis.^11^ In 2015, Wu *et al.*^12^ reported on an RBC micromotor fabricated by encapsulating magnetic nanoparticles (MNP) inside cells using hypotonic solutions to induce pore swelling, allowing for the passage of MNPs into cells. The iron oxide nanoparticles were used to induce net magnetization of the micromotor, allowing for alignment *via* magnetic field and propulsion by application of an acoustic field, utilizing asymmetric distributions of nanoparticles. Later, Pan *et al.*^13^ created micromotors by altering shape of RBCs with a hypertonic solution resulting in a bowl shape which was subsequently coated with platinum and actuated *via* air bubbles produced using hydrogen peroxide as a catalyst. RBC-based micromotors also can be manufactured by packing indocyanine green (ICG) and hemoglobin particles inside the RBC membrane.^14^ The synthesized micromotors were explored for photodynamic cancer therapy and their propulsion capability was demonstrated *via* magnetic and acoustic fields. Recently, RBC mimetic micromotors have been used as cargo carriers for tumor therapy and imaging applications that could be actuated *via* a magnetic field. Hou *et al.*^15^ investigated synthetic fabrication methods of biomimetic micromotors and actuated them using a rotating magnetic field causing them to roll along the surface.

Many envisioned applications for RBC micromotors, especially in therapeutics, involve navigation in bulk fluids, such as the blood stream. The ability to control the navigational dynamics of these micromotors in bulk fluids is of significant value but is also highly challenging due to the absence of symmetry breaking factors. At low Reynolds numbers if a micromotor executes geometrically reciprocal motion, then the net displacement of the micromotor must be zero if the fluid is incompressible and Newtonian – an effect captured in the Scallop theorem.^16^ Natural microorganisms have developed various strategies to overcome this effect, such as the periodic forcing of flagella or cilia which enable swimming at low Reynolds number by exploiting the friction along the elastic flagellum to break time-reversibility or the constant corkscrew motion of bacterial flagella which creates a helical traveling wave. Synthetic counterparts to both the elastic flagellum and the corkscrew have been extensively studied using actuation mechanisms such as the magnetic and electric fields.^17^ While RBCs seemingly lack the asymmetry required to achieve propulsion at low Reynolds numbers, we find that the inherent shape anisotropy in combination with rotating magnetic fields is sufficient to realize swimming modes both along surfaces and in the bulk fluid.

Here we report a new technique for fabricating erythrocyte-based magnetic micromotors involving a streptavidin-biotin bonding between the magnetic microparticles and the RBCs that allows attachment to the cell surface without a need for modifying the membrane pore size. Compared with direct encapsulation methods which utilize osmotic pressure differences that can rupture cell membranes for magnetic microparticle loading, biochemical surface attachment of magnetic particles to the outer membrane of the RBCs offers a more controllable self-assembly method of magnetic functionalization without significantly affecting native surface properties of cells such as pore size and permeability. Additionally, the presence of the microparticles on the surface contributes towards the shape-anisotropy necessary to achieve swimming in bulk Newtonian fluids. We find that the streptavidin-biotin bonding greatly enhances the formation of micromotors as compared to non-specific adhesion. Biotin is a small molecule which includes an amide group (CONH_2_) and multiple hydrogen bond donors. The streptavidin protein has four binding sites, each of which has multiple amino acid residues with hydrogen bond acceptor sites. As a result, several hydrogen bonds are formed between the two when they bind.^18^ The biotin-streptavidin hydrogen bonds exhibit large free energy,^19^ providing binding stabilization. Additionally, hydrophobic bonds also form between biotin and streptavidin as each streptavidin is composed of several beta strands twisted together and forms a ‘beta-barrel structure’ with hydrophobic groups in those pockets,^20^ which provide binding sites for the hydrophobic residues of biotin. The combined contributions of these non-covalent bonds results in a specific binding that is stable across a wide range of pHs and temperatures and has a dissociation constant in the order of 10^−15^ M.^21^ We demonstrate the ability to control their motion using a single uniform rotating magnetic field through two different motion mechanism – rolling along the surface through proximity-induced symmetry breaking and swimming in the bulk fluid. The attachment of the magnetic beads and the natural deformability of the RBC creates shape anisotropy that allows for these micromotors to convert rotational motion into translation under a rotating magnetic field in bulk fluid similar to other achiral synthetic swimmers.^22,23^ We describe swimming and rolling kinematics of the micromotors including velocity and step-out frequencies for both translation modes in PBS and serum. Finally, we demonstrate the ability to control the directionality of the micromotors using external magnetic fields both close to the substrate and in bulk fluid.

## Experimental

### Erythrocyte-based biohybrid micromotor fabrication

First, RBCs were isolated from whole bovine blood (Carolina Biological Supply Company, Item#: 828514). Prior to use, cells were washed three times with ice-cold phosphate-buffered saline (PBS) to remove leukocytes, platelets, and plasma from the solution and in order to reduce potential allergic reactions due to contaminating proteins.^30,31^ The washed RBCs were then resuspended in fresh ice-cold PBS and treated by biotin (Thermo Scientific, #21338). The resulting suspension was mixed well and allowed to incubate at 4 °C overnight. Following the incubation period, the biotinylated RBCs were washed three times with ice-cold 1 × PBS to remove excess biotin. Streptavidin-coated magnetic beads (0.21 μm in diameter; Spherotech Inc., #SVM-025-5H) were mixed with the biotinylated RBCs suspension in PBS to create the final biotinylated RBCBMs.

### *In vitro* combability

To assess the biocompatibility of RBCB micromotors, MTT assays were performed. HEK-293 cells were cultured in 96-well plates, with a seeding density of 1 × 10^4^ cells per well and incubated for 24 hours at 37 °C with 5% CO_2_. Following a 48 h incubation, 1 × 10^4^ RBCs and 1 × 10^4^ RBCB micromotors were added and co-cultured with HEK cells for 24 hours and 72 hours separately. After incubation, cell culture media was decanted, and cells washed with 1 × PBS. A mix of 90 μL serum free media and 10 μL MTT solution (5 mg mL^−1^ in PBS) was added and incubated with cells for 2 hours. After removing MTT, dimethyl sulfoxide was added to each well and mixed thoroughly. The absorbance of the suspension was measured with 595 nm light. Cell viability was calculated by:
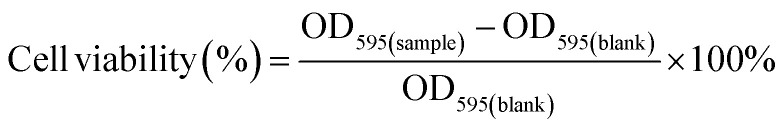


OD_595(sample)_ and OD_595(blank)_ represent the absorbance values of the testing samples and the blank control, respectively.

## Results and discussion

### Fabrication of erythrocyte-based biohybrid micromotors

**Fig. 1:**
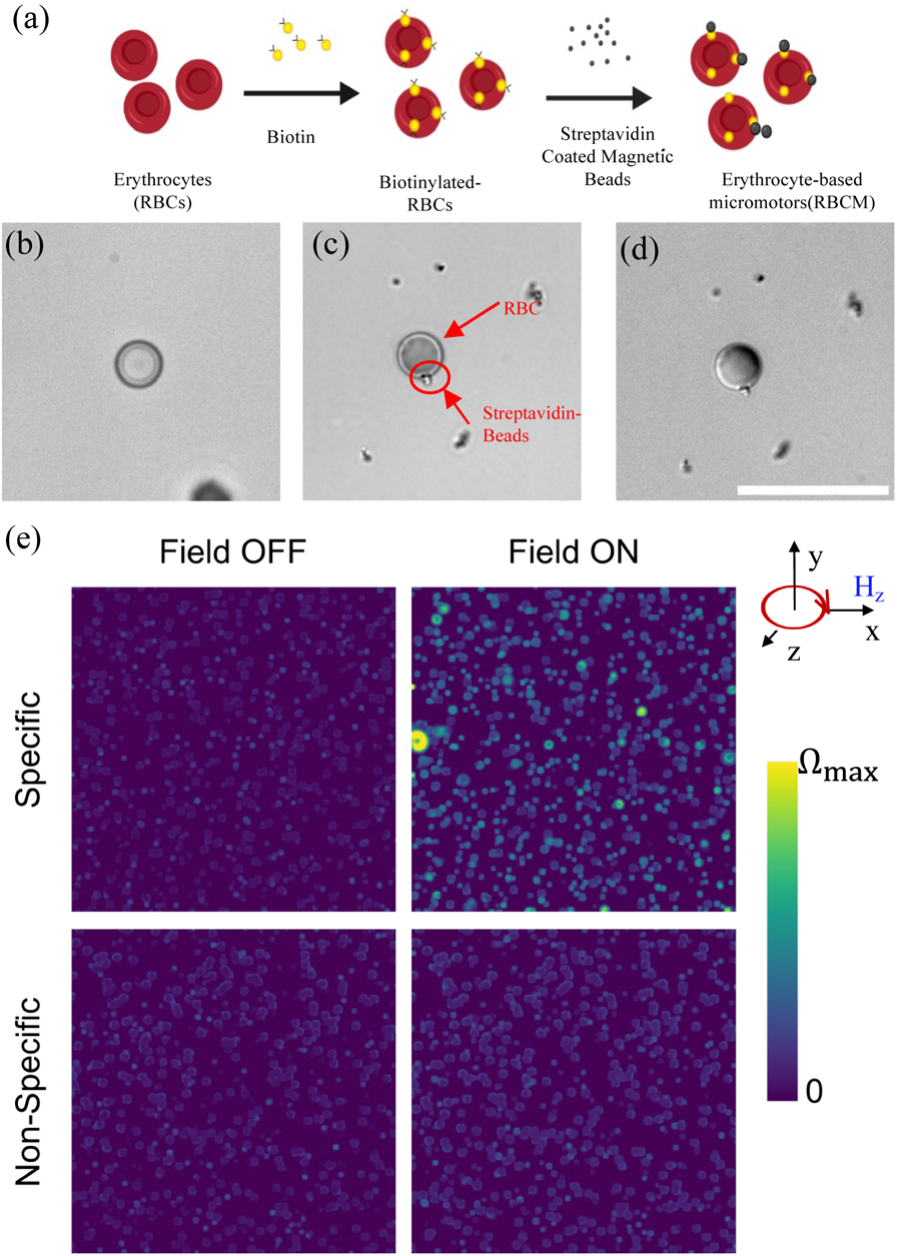
(a) Schematic of erythrocyte (RBC)-based biohybrid micromotors (RBCBMs) fabrication. (b) Bright field images of RBCs. Images of RBCBM under (c) bright field, (d) differential interference contrast (DIC); 60× magnification, scale bar = 20 μm. (e) Velocity magnitude of the RBCs and RBCBMs with (specific MNP binding) and without (non-specific MNP binding) biotin in the absence and presence of an external in-plane rotating magnetic field.

The synthesis of red blood cell biohybrid micromotors (RBCBM) was achieved by utilizing the remarkable strength of the biotin-streptavidin interaction. Sulfo-NHS-LC-LC biotin, through its NHS ester, binds to the amine groups on RBC membrane surface proteins forming non-covalent amide linkages. Biotin is negatively charged due to the sodium sulfonate group on its succinimidyl ring, and thus to the biotin cannot permeate the negatively charged cell membrane.^24^ Therefore, only exposed primary amines are biotinylated. Biotin treated RBC membranes were bound to streptavidin-coated magnetic beads (210 nm) to form RBCB micromotors (Fig. 1a). Magnetic beads adhering to RBCs are clearly visible, in contrast to untreated RBCs (Fig. 1b). RBCBMs retain the characteristic shape of RBCs throughout the attachment process, with an overall average diameter of approximately 7 μm. In order to confirm the magnetization of RBCBMs we suspended the cells obtained after the above process in an experimental cell composed to two clean glass slides separated by a PDMS spacer. The RBCBMs quickly settle towards the bottom substrate as they are density mismatched and maintain a constant height above the surface. We then applied an in-plane rotational magnetic field *H*_*z*_(*t*) = *H*_o_[sin(2π*ft*)*e*_*x*_ + cos(2π*ft*)*e*_*y*_] (*H* = 10 mT, *f* = 4 Hz) and recorded the magnetic response of the RBCBM (Video S1†). As a control, we performed the same experiments for RBCs and magnetic beads incubated together for the same time period as RBCBMs, but without biotin functionalization. In order to characterize the magnetic response, we performed optical flow analysis on the obtained videos using the Farneback algorithm^25^ implemented in OpenCV, which allows us to estimate the displacement of each pixel between consecutive frames. In Fig. 1e we plot the velocity magnitude with and without applied field for both cases of biotinated and non-biotinated cells. We observed a strong magnetic response in the case of biotinated cells compared to our control, confirming a greater coupling between the cells and magnetic microparticles promoted by the specific biotin-streptavidin bonding. To investigate the *in vitro* biocompatibility of RBCB micromotors, a set of MTT assays were performed. The results show that HEK cell viability was above 90% in all groups co-cultured with RBCs or RBCBMs for 24 h or 72 h (as Fig. SI†). The RBCB micromotors did not show significant toxicity to mammalian cells.

### Propulsion of RBCB micromotors

In order to investigate the motion of the RBCBM, a magnetic field generator (MagnebotiX, MFG-100) was used. The MFG-100 system uses electromagnets with ferromagnetic cores to produce magnetic fields in a spherical workspace of approximately 10 mm in diameter, allowing us to generate varying strengths and frequencies of the rotating magnetic field.^26,27^ The MFG system was controlled using custom MATLAB® scripts. The parameters of the rotating magnetic field in our experiments are expressed by magnetic field strength (*H*), frequency (*f*), time (*t*) and the unit vectors along the *x*, *y* and *z* axes (*e*_*x*_, *e*_*y*_, and *e*_*z*_). The rotating magnetic fields used in the experiments are in the *x*–*z* plane (with the *y*-axis as the central axis) and the *y*–*z* plane (with the *x*-axis as the central axis), which are described as:

1 In the *x*–*z* plane: *H*_*y*_(*t*) = *H*_o_[sin(2π*ft*)*e*_*x*_ + cos(2π*ft*)*e*_*z*_]

2 In the *y*–*z* plane: *H*_*x*_(*t*) = *H*_o_[sin(2π*ft*)*e*_*y*_ + cos(2π*ft*)*e*_*z*_]

RBCBMs were suspended in saline buffer (PBS, pH 7.4) solution or serum. Within our experimental cell we found a distribution of cells both in the bulk fluid and close to the bottom substrate. The surface of RBC cell membranes and Sulfo-NHS-LC-LC biotin are both negatively charged. Therefore, fabricated RBCB micromotors share this negative charge characteristic. In experiments, the substrate slides were coated with bovine serum albumin (BSA), which is likewise negatively charged.^28^ Consequently, even when the RBC settles to the bottom of the suspension, they resist adhering to substrate due to electrostatic repulsions. During motility experiments, it was also observed that RBCB micromotors didn't adhere to the substrate even when they were actuated close to the glass surface. When a rotating magnetic field was applied, we found two different modes of motion of the RBCBMs depending on the distance between the RBCBM and the surface of the experimental cell. For the RBCBMs that settle to the bottom of the suspension and are near the bottom plane (approximately 10–20 μm) we found that they rotate around their central axis and roll along the direction perpendicular to the field rotation axis under the torque generated by the uniform magnetic field (Video S2†). With an external magnetic field intensity *H*, the magnetic torque applied to the RBCB micromotor is given by *N*_*m*_=*V*·*m* × *B*, where *V* is the volume of magnetic material, *m* is the internal magnetization, *B* is the magnetic density, *B* = *μ*_o_·(*H* + *M*); *μ*_o_ is magnetic permeability and *M* represents magnetization. The proximity of the surface to a rotating RBCBM creates a frictional asymmetry causing the structures to roll along the surface, similar to what has been observed for other magnetic micromotors.^15^

At the same time, some RBCBMs were observed further from the bottom surface, in the bulk fluid (>50 μm from substrate). When the same rotating magnetic field was applied, a different propulsion mode was observed where the RBCBMs start to rotate and swim along the direction of the axis of the rotating magnetic field (Video S3†). The swimming of the RBCMs in the bulk has a completely different mechanism of motion from the rolling observed near a wall. For the RBCBMs swimming in the bulk, surface friction is negligible due to the large distance between the RBCBM and bottom plane. In Stokes fluid flows, the mobility matrix of the RBCBM can be expressed as:^22,23,29^3
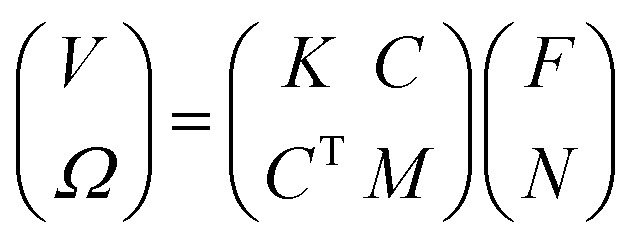
where, *V* and *Ω* represents translational and rotational velocity respectively; *F* and *N* respectively are the force and torque applied on the RBCBM and *K*, *M*, *C* are submatrices that are related to translational and rotational velocity, force, and torque. Because the RBCBM was propelled by a single uniform rotational magnetic field, we can assume *F* = 0. Velocities of the RBCBM can then be rewritten as:

4 *V* = *C*·*N*

5 *Ω* = *M*·*N*

The structure of a RBCBM is not axisymmetric nor does it have more than three perpendicular symmetry planes, leading to *C* ≠ 0,^22^ which allows the micromotor to have translational velocity in a bulk fluid. Due to the different actuation mechanisms, these two kinds of RBCBM motion modes result in perpendicular translational directions with respect to each other when the same rotating magnetic field is applied.

**Fig. 2:**
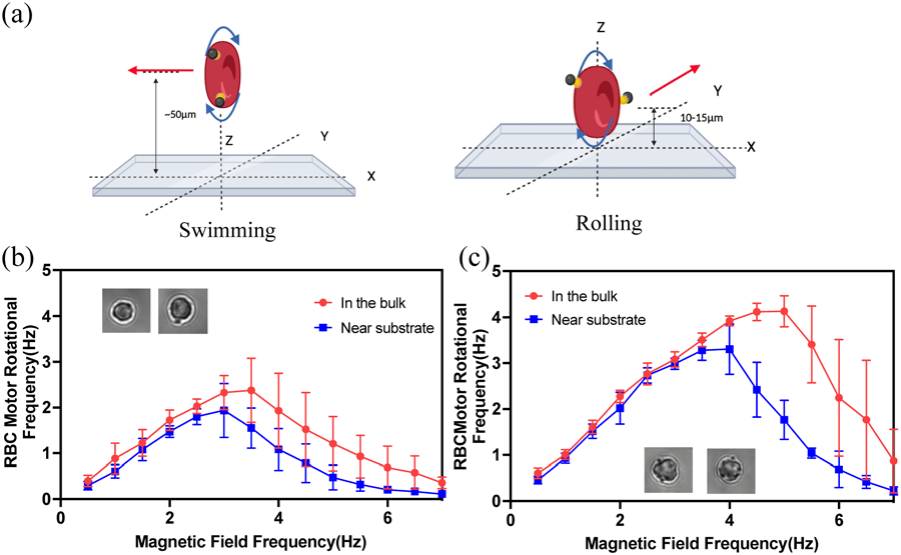
(a) Schematics of RBCBM swimming in the bulk (left) and rolling on the substrate (right). The step-out frequencies of each RBCBM were obtained from the experimental data of five randomly selected RBCMs (*n* = 5 for all groups). Average RBCBM rotational frequencies at different rotational magnetic field frequencies (b) RBCBM with one/two surface-attached microbead (c) RBCBM with three or more surface-attached microbeads.

Next, we measured the step-out frequency of RBCBMs to find the optimum actuation frequency of the magnetic field applied to the micromotor. When a rotating magnetic field is applied on a magnetized particle, the particle rotates synchronously with the magnetic field, so long as the magnetic torque is greater than the hydrodynamic drag acting upon the particle. When the angular velocity of the magnetic field is increased beyond a critical value, the force of the hydrodynamic drag dominates, restricting the particles rotation and resulting in decreased rotational speed, as the particle is no longer able to rotate at the same frequency as the magnetic field. This critical frequency value is an inflection point between synchronous and asynchronous rotation, known as the ‘step-out frequency.’ The RBCBMs will theoretically reach their maximum rotational and translational velocities when the magnetic field rotates at this frequency, therefore the step-out frequency of various RBCBMs were determined and used in subsequent experiments. Here, a uniform rotational magnetic field (10 mT) in *x*–*z* plane (*H*_*y*_) was consistently applied. The magnetic field frequency was gradually increased from 0.5 Hz, in 0.5 Hz steps, until a significant decrease in the rotational frequency of the micromotor was observed. Finally, the experiment was stopped at a frequency of 7 Hz. In the experiments, we found that RBCBMs have different compositions of bound magnetic particles. RBCBMs were observed to have mostly one, two, or three beads attached. Their response under a rotating magnetic field is not identical; in the case of RBCBMs which have multiple magnetic particles attached to the cell, additional torque is generated by the magnetic material enables synchronous actuation at higher frequencies under the same magnetic field. Therefore, RBCBMs were classified into two groups: RBCBMs with one or two beads attached and RBCBMs with three or more beads attached, representative of the typical distribution found in our system. From Fig. 2 we conclude that for both RBCBM in the bulk solution and near the substrate, the RBCBMs with three or more microbeads attached have higher step-out frequencies than those with only one or two microbeads attached. More magnetic beads attached provide a stronger drive force under the magnetic field, which results in a greater step-out frequency. Moreover, we also found that apart from the number of attached magnetic particles, the motion mechanism of RBCBMs affects the step-out frequency as well: the step-out frequency of the swimming RBCBMs was observed to be higher than that of rolling ones (Fig. 2). We attribute this difference to the lack of the effect of surface friction for bulk swimmers, allowing them to synchronous rotate at higher actuation frequencies.

**Fig. 3:**
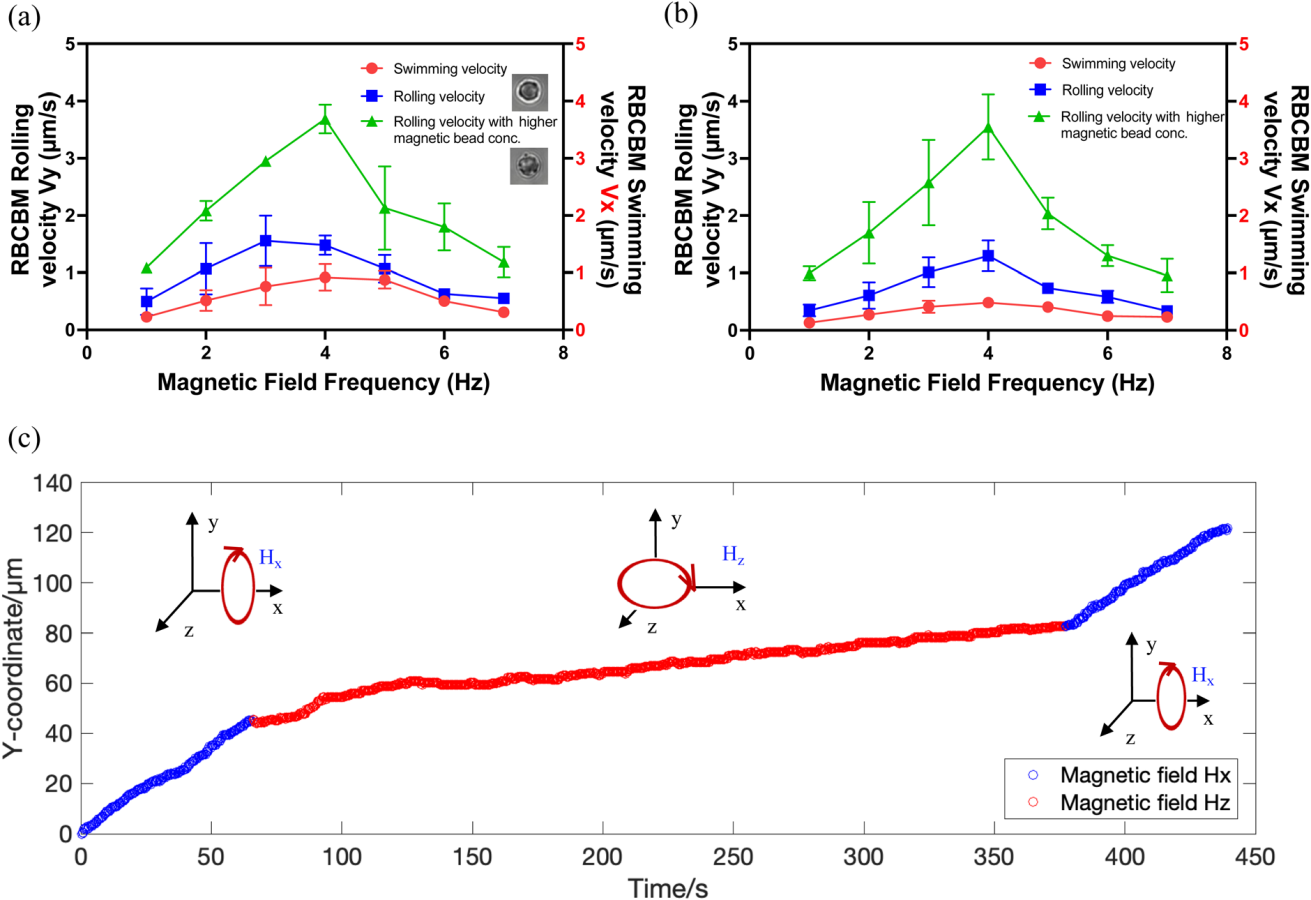
RBCBM velocity of two motion modes with respect to magnetic field frequency under a 10 mT rotating magnetic field, in (a) saline buffer (PBS); (b) Bovine serum, *n* = 5. (c) Plot shows the change of the RBCBM *y*-coordination in PBS, at 4 Hz, 10 mT. Blue dots represent RBCBM motion with a magnetic field perpendicular to the *x*-axis (*H*_*x*_) on, allows RBCBM rolling in *y*. Red dots represent the RBCBM motion with an in-plane magnetic field (*H*_*z*_).

Rolling and swimming velocities were determined for a range of driving frequencies (Fig. 3a). With similar results to the previous measurement of step-out frequencies (Fig. 2), the rolling RBCBMs differ in their rolling speed because of the different number of surface-attached magnetic beads. Therefore, we classified RBCBM rollers into those with 1–2 beads attached and those with 3–4 beads attached to plot the speed profiles. It should also be noted that for RBCBM swimmers we rarely observed motors with only one attached magnetic particle capable of swimming. A few RBCBMs with two beads were able to swim, although most were not. However, most RBCBMs with three or more attached beads were capable of swimming. We found that when the magnetic field frequencies are lower than the step-out frequency, the translation velocity of the RBCBM increases approximately linearly for both rolling and swimming modes. The RBCBM rolling velocities were greater than the swimming velocity for the same driving frequencies and amplitudes due to the higher efficiency of the rolling mechanism compared to the swimming mechanism. The magnetic field rotational frequencies that allow RBCBM swimmers or rollers to reach their maximum translational speed are aligned with their step-out frequencies. For *in vivo* RBCB micromotor biomedical applications, the ability to efficiently propel in complex biological fluids will be critical. Therefore, after obtaining the velocity profile of the RBCBM in a low-viscosity saline buffer solution (PBS, ∼1 mPa s), we also performed velocity sweeps of the RBCBMs in bovine serum (∼5 mPa s). Compared to the velocity in PBS, in serum the velocity of the RBCBM swimming in the bulk decreased most significantly, approximately 50%, as shown in Fig. 3b. The RBCB micromotors with 1–2 MNPs attached also showed a slight decrease of rolling velocity near the substrate; those with more than 3 MNPs attached exhibited almost the same rolling velocity in serum as in PBS. Although the micromotors motion is slightly attenuated in serum, the ability to translate in this fluid provides a foundation for future investigations targeting *in vivo* applications.

Furthermore, the stability of RBCB micromotors under the action of a sustained magnetic field is another important concern for biomedical applications. Even though the interaction between biotin and streptavidin is the strongest recognized non-covalent biological effect, there is still a concern about the detachment of MNPs from the surface of RBCs under long-term action of a magnetic field. To address this, we designed an experiment where RBCB micromotors were exposed to a rotating magnetic field of 10 mT, 4 Hz over several minutes. Initially, a rotational magnetic field perpendicular to the *x*-axis (*H*_*x*_) was applied which enables micromotor rolling in the *y* direction, and after 50 s the field was turned off. Subsequently an in-plane rotational magnetic field (*H*_*z*_) was applied for five minutes which rotates the micromotors in-place on the glass substrate. Finally, a rotational magnetic field (*H*_*x*_) was applied again for 50 s. The resulting trajectory of a RBCBM is shown in Video S4.† The evolution of RBCBM *y*-coordinate position over time is plotted in Fig. 3c. The positions shown in blue indicate translational rolling of the RBCBM before and after the continuous in-plane rotation, shown in red. A linear fit shows that the rates of the RBCB micromotor before and after in-plane rotation (red) were 1.45 μm s^−1^ and 1.44 μm s^−1^, respectively. The number of magnetic particles attached to the RBCBM is correlated to its rolling speed (Fig. 3c), thus the constant rolling rate before and after the magnetic field action indicates that no particles were detached during magnetic actuation.

### Steerability of RBCB micromotors

**Fig. 4:**
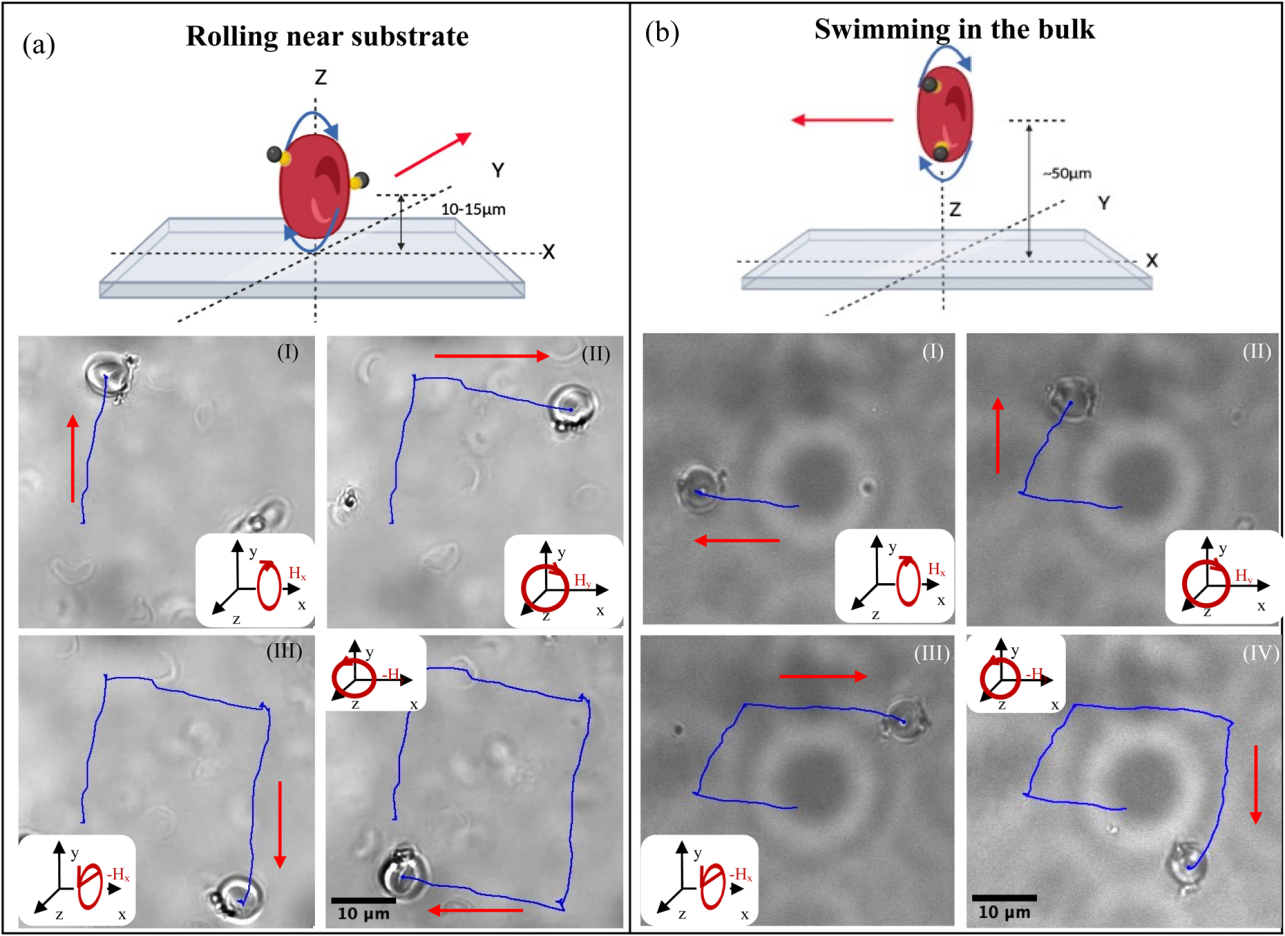
RBCBM actuated in a square path with a 10 mT rotating magnetic field, frequency equals to each step out frequencies. (a) RBCBMs rolling on a substrate (∼10 μm from the substrate); (b) RBCBM swimming in bulk fluid (∼50 μm from the substrate). Under a same orientation rotating magnetic field, RBCM bulk swimming directions and rolling directions are always orthogonal to each other.

In addition to determining the dynamic properties of the RBCB micromotors, we conducted experiments aimed at demonstrating RBCBM maneuverability and demonstrating the difference between the two modes of motion. As in previous experiments, the strength of the magnetic field was kept constant (10 mT) and the frequency of the magnetic field applied was selected to be step out frequency of the respective RBCBM. All steering was achieved by successively changing the rotation direction of the magnetic field. At the step out frequency we found that in both translational modes of swimming and rolling, the RBCBMs respond immediately to the change in the applied magnetic field direction (as Fig. 4 and Video S5, S6†). As expected, the trajectories of the rolling RBCBMs are perpendicular to trajectories of bulk swimming for the same applied directional magnetic field changes. This allows for precise control over the location of the RBCBMs in micro-environments and demonstrates the ability to maneuver them along complex paths on-the-fly.

## Conclusions

In summary, erythrocyte based biohybrid micromotors were fabricated by linking streptavidin-coated magnetic beads to the surface of erythrocytes. RBCB micromotors were characterized with respect to their step-out frequency, velocity profiles, and steerability were investigated. The RBCB micromotors were actuated using a uniform 10 mT rotating magnetic field and exhibited two modes of movement, rolling and swimming, depending on their proximity to the bottom surface. The maximum rotational frequency of the RBCBM is dependent on the number of attached magnetic particles and the motion of RBCBMs. The number of beads attached to RBC surface increases the step out frequency. Also, RBCBM swimmers have a higher step-out frequency when swimming than when rolling. As shown through these experiments the translational velocity of the RBCBMs, both when swimming and rolling, increases with the rotational frequency of the magnetic field, although the RBCBMs being actuated in bulk solution are slower than those rolling on surfaces. Moreover, the ability of the RBCBM to propel by swimming and rolling modes in a biofluid, serum, was also demonstrated. It was also shown that the RBCB micromotors were capable of following set pathways as determined through an open loop computer control mechanism. These experiments demonstrate that RBCBMs can be precisely controlled for both swimming and rolling using a single rotating magnetic field, both simplifying the control input required for actuation, introducing possibilities for switching between different modes of translational motion dependent on the location of the micromotors with respect to environmental boundaries. The biosafety of the RBCB micromotor was also demonstrated, with no significant cellular toxicity within 72 hours. It was also demonstrated that magnetic microbeads do not detach from the RBC membrane when driven by a magnetic field even over long periods of time, which provides the possibility for biomedical applications. In the future, RBCBMs from autologous sources could be used *in vivo* as actively driven carriers of therapeutic compounds or for transportation of medical imaging contrast agents. Loading of theranostic agents within RBCBMs can be achieved through well-established passive diffusion-based methods that utilized hypotonic solutions that expand the cellular membrane opening pores for loading. Overall, our work shows the propulsion ability of the RBCBM with two different propulsion modes in different fluid types with a single uniform rotational magnetic field and also demonstrates their viability in the presence of biological cells paving the way for their potential applications in personalized medicine.

## Author contributions

Qi Wang: writing – original draft, writing – review & editing, visualization, validation, methodology, investigation, software, formal analysis, data curation. Sophie Jermyn: writing – original draft, writing – review & editing, visualization. David Quashie Jr.: data curation, methodology, formal analysis, software. Jaideep Katuri: writing – review & editing, methodology, visualization. Sarah Elizabeth Gatti: writing – original draft. Jamel Ali: writing – review & editing, visualization, methodology, investigation, visualization, supervision, project administration, funding acquisition.

## Conflicts of interest

There are no conflicts to declare.

## Supplementary Material

RA-013-D3RA05844A-s001

RA-013-D3RA05844A-s002
