# Supplementary material for: Magnetically actuated swimming and rolling erythrocyte-based biohybrid micromotors
Source: RSC Adv. 2023 Oct 23;13(44):30951–8. doi: 10.1039/d3ra05844a (PMC10591291; doi:10.1039/d3ra05844a)
Supplement: RA-013-D3RA05844A-s002 [file RA-013-D3RA05844A-s002.pdf]

Electronic Supplementary Material (ESI) for RSC Advances.
This journal is © The Royal Society of Chemistry 2023

Supporting Information

Magnetically Actuated Swimming and Rolling Erythrocyte-based Biohybrid Micromotors

Qi Wang,*[ab] Sophie Jermyn,[ab] David Quashie Jr.,[ab] Elizabeth Gatti,[bc] Jaideep Katuri,[ab] and Jamel Ali[ab]

[a)] *Department of Chemical and Biomedical Engineering, FAMU-FSU College of Engineering, Tallahassee, Florida 32310, USA.*
[b)] *National High Magnetic Field Laboratory, Tallahassee, Florida 32310, USA.*
[c)] *Department of Biomedical Engineering, Vanderbilt University College of Engineering, Nashville, Tennessee 37235, USA.*

(*Email: jali@eng.famu.fsu.edu)

Supplementary Video 1: The RBCs and RBCBMs with (specific MNP binding) and without (non-specific MNP binding) biotin in the absence and presence of an external rotating magnetic field. The external field strength is 10mT rotating in the x-y plane, 4Hz.

Supplementary Video 2: Magnetically actuated rolling of red blood cell micromotors (RBCMs) in a 1X PBS solution near an underling substrate. The external field strength is 10mT rotating in the y-z plane.

Supplementary Video 3: Magnetically actuated bulk swimming of red blood cell micromotors (RBCMs) in a 1X PBS solution ~50 µm away from a substrate. The external field strength is 10mT rotating in the y-z plane.

Supplementary Video 4: Demonstration RBCB micromotor magnetic stability under sustained external field actuation. The external magnetic field strength is 10mT, 4Hz. First, the external field is set to rotate in the y-z plane for 50 sec, resulting in RBCBM rolling near the substrate. Then, the field is set to rotate in the x-y plane for 5min. Finally, magnetic field is set to again rotate in the y-z plane for 50 sec.

Supplementary Video 5: Open loop control of RBCBM rolling near interface under a 10mT 4Hz magnetic field.

Supplementary Video 6: Open loop control of RBCBM swimming in bulk fluid under 10mT, 4Hz magnetic field.

Supplementary Figure:

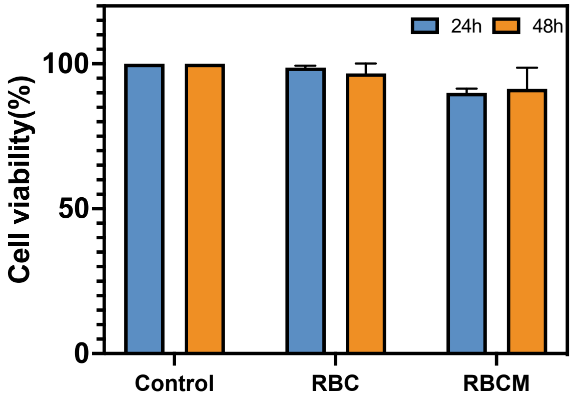

To investigate the *in vitro* biocompatibility of RBCB micromotors, a set of MTT experiments were performed. The results indicate that HEK-293 cell viability was above 90% in all groups, including HEK-293 cells co-cultured with RBCs or RBCBMs for 24 and 72 hrs.
